# Effects of oral macimorelin on copeptin and anterior pituitary hormones in healthy volunteers

**DOI:** 10.1007/s11102-021-01132-9

**Published:** 2021-02-22

**Authors:** Sandrine A. Urwyler, Sven Lustenberger, Juliana R. Drummond, Beatriz Santana Soares, Deborah R. Vogt, Nicola Ammer, Kevin C. J. Yuen, Antonio Ribeiro-Oliveira, Mirjam Christ-Crain

**Affiliations:** 1 Department of Endocrinology, Diabetology and Metabolism, University Hospital Basel, Petersgraben 4, 4031 Basel, Switzerland; 2 Department of Clinical Research, University Basel, Basel, Switzerland; 3 Faculdade de Medicina da UFMG, Universidade Federal de Minas Gerais, Belo Horizonte, Brazil; 4 Department of Clinical Research, Clinical Trial Unit, University Hospital Basel, University of Basel, Basel, Switzerland; 5 Aeterna Zentaris GmbH, Frankfurt, Germany; 6 Department of Neuroendocrinology, Barrow Neurological Institute, University of Arizona College of Medicine and Creighton School of Medicine, Phoenix, AZ USA

**Keywords:** Ghrelin agonist, Macimorelin, Copeptin, Pituitary hormones, Diabetes insipidus

## Abstract

**Purpose:**

The test with the highest diagnostic accuracy for diabetes insipidus is copeptin measurement after hypertonic saline infusion. However, the procedure is cumbersome and unpleasant due to rapid sodium increase. An oral stimulation test would be highly desirable. Macimorelin, an oral ghrelin agonist, is a newly approved diagnostic test for growth hormone (GH) deficiency, but its effects on copeptin/vasopressin are unknown and the effects on other pituitary hormones only scarcely investigated.

**Methods:**

In this prospective, interventional, proof-of-concept study Copeptin and anterior pituitary hormones were measured in 28 healthy volunteers on two test days at baseline, 30, 45, 60, 90 and 120 min after a single dose of macimorelin (first visit: 0.5 mg/kg, second visit: 0.75 mg/kg).

**Results:**

Baseline copeptin levels were 5.26 pmol/L [1.57, 6.81] and did not change after macimorelin intake (0.5 mg/kg: maximal median change 0.40 [− 0.49, 0.65] pmol/L, p = 0.442; 0.75 mg/kg: − 0.13 [− 0.45, 0.17] pmol/L, p = 0.442. Median GH levels increased from 3.67 mU/L with a maximal median change of 94.66 [IQR 56.5; 110.96] mU/L, p < 0.001. No effect was seen on cortisol, ACTH, LH and FSH levels. Prolactin (max. median change 100 [2.5; 146.5] mU/L, p = 0.004) and free thyroxine (fT4) (0.5 [0.2; 0.8] pmol/L, p < 0.001) increased, whereas TSH decreased (− 0.18 [− 0.22, − 0.09] mU/L, p < 0.001).

**Conclusion:**

We confirm an increase of GH upon macimorelin in healthy volunteers. However, macimorelin did not stimulate copeptin and therefore does not provide an oral test alternative for the diagnosis of diabetes insipidus. Additionally, a stimulatory effect was seen for prolactin and fT4, but not for ACTH and gonadotropic hormones.

**Registration:**

The trial was registered on ClinicalTrials.gov (NCT03844217) on February 18, 2019.

## Introduction

The diagnosis of pituitary hormone deficiencies is often cumbersome as an intravenous application of the stimulatory agent is mandatory [1]. In particular, the differential diagnosis of diabetes insipidus is challenging [2] and to date the test with the highest diagnostic accuracy is copeptin measurement after hypertonic saline infusion [3]. However, this test requires an intravenous application of the hypertonic saline solution, and close medical supervision throughout the test for tight control of plasma sodium levels. Moreover, it often causes discomfort in patients due to the rapid increase in plasma sodium levels. Recently, arginine infusion—well known as a growth hormone secretagogue (GHS)—was found to be a potent stimulator of the neurohypophysis and provides a new diagnostic tool in the differential diagnosis of central diabetes insipidus [4]. However, despite its better tolerance, this test still needs intravenous administration. An oral stimulation test would be easier to perform, cause less risks and discomfort to the patient, and would require fewer resources in clinical practice.

A single oral dose of macimorelin—a ghrelin receptor agonist—has been shown to stimulate GH-secretion and has proven as a well-tolerated, reproducible and safe diagnostic test for the diagnosis of adult growth hormone deficiency (AGHD) in comparison to the insulin tolerance test (ITT) and the GHRH-arginine test [5, 6]. Ghrelin has a stimulatory effect on copeptin/vasopressin both in vitro and in rodents [7–10]. Other GHS such as hexarelin [11] and arginine infusion [4] stimulate copeptin/vasopressin in humans. In analogy to this, we hypothesized that macimorelin would stimulate copeptin secretion from the posterior pituitary gland.

Not only the diagnostics of posterior pituitary hormone deficiencies are difficult, but also the assessment of anterior pituitary hormones can be challenging. Standard diagnostic tests of adrenal insufficiency are the ITT or cosyntropin test in cases with indeterminate morning cortisol levels to confirm the diagnosis of adrenocorticotropic hormone (ACTH) deficiency [12], and for this condition, there is no available oral stimulatory test. Growth hormone-secretagogues (GHS) are also known to stimulate the hypothalamo-pituitary-adrenal (HPA)-axis, and previous data have shown a stimulatory effect of hexarelin—a GHS, which is administered intravenously, on ACTH and cortisol in healthy volunteers [11]. We therefore hypothesized that macimorelin would stimulate also the HPA-axis and other hormones of the anterior pituitary gland apart from the known effect on GH.

## Participants and methods

This study was conducted as a single-centre, open-label, prospective interventional proof-of-concept study in compliance with the ethical principles based on the Declaration of Helsinki and the applicable International Conference on Harmonization (ICH) guidelines on good clinical practice. Local ethic committees and Swissmedic approved the study protocol. Written informed consent was obtained from all study participants before inclusion. The study was preregistered on ClinicalTrials.gov (NCT03844217).

### Study participants

Study participants had to be healthy and were recruited as volunteers via email at the University of Basel. Inclusion criteria were age between 18 and 60 years and no regular medication apart from hormonal contraception in females. Exclusion criteria were any known known medical history or underlying health condition, particularly any chronic renal or liver disease, body mass index (BMI) ≤ 18.5 kg/m^2^ or ≥ 40 kg/m^2^, pregnancy or breastfeeding, evidence of drinking disorders and diuresis (defined as polyuria > 50 mL/kg body weight/24 h and polydipsia > 3 l/24 h), a prolonged QT interval (QTc > 500 ms) or a known allergy towards macimorelin.

### Study drug

Macimorelin acetate is an oral ghrelin full receptor agonist, which was provided by Aeterna Zentaris GmbH. It has a molecular weight of 535.6 g/mole and its solubility in water is 300 mg/mL. Its structural formula has been published previously [13].

### Study procedure

A study physician screened all participants in a screening visit. Eligibility criteria were checked, an electrocardiogram (ECG) to exclude long QT was conducted, a medical history questionnaire was obtained and a pregnancy test was performed in all women.

Study participants attended two study visits (V1 and V2) scheduled in the morning after an overnight fast. Participants were allowed to drink until midnight, alcohol was not allowed 24 h before the test started. At V1 the test was performed with a single oral dose of 0.5 mg/kg body weight macimorelin, which is the dose approved in the United States and Europe to diagnose AGHD [6]. The second test day V2 was scheduled at least one week apart using a single dose of 0.75 mg/kg body weight macimorelin. A peripheral intravenous catheter was placed 30 min before the baseline blood examination. Blood samples were drawn at baseline (0 min) and 30, 45, 60, 90 and 120 min after the intake of macimorelin. Vital signs were recorded at the same point of time when blood was drawn. An ECG to assess QTc-interval was performed at baseline and at 120 min of each study visit.

### Laboratory assessment

All laboratory analyses were performed at the centre laboratory at University Hospital Basel immediately after collecting the samples. Copeptin levels were measured with a chemifluorescence sandwich immunoassay (BRAHMS CT-proAVP KRYPTOR, from BRAHMS GmbH, Hennigsdorf, Germany), with a lower detection limit of 0.4 pmol/L.

ACTH was quantified with chemiluminescence immunoassay (ACTH Immulite, Siemens Healthcare Diagnostics Products Ltd., Gwynedd, UK), with a reference range < 46.0 pg/mL after collecting the blood on ice. Cortisol, Prolactin, free thyroxine level (fT4), thyroid stimulating hormone (TSH), GH, luteinizing hormone (LH) and follicle stimulating hormone (FSH) were determined with an electrochemiluminescence immunoassay (ECLIA) (Cobas8000, Roche Diagnostics GmbH, Mannheim, Germany) and insulin like growth factor-1 (IGF-1) levels were measured with a chemiluminescence immunoassay (CLIA) (LIAISON XL, DiaSorin S.p.A., Saluggia, Italy).

### Study endpoints

The predefined primary endpoint was the maximal change in copeptin levels upon the intake of a single oral dose of macimorelin 0.5 mg/kg body weight. Predefined secondary endpoints were the maximal change of copeptin levels upon intake of a single oral dose of macimorelin 0.75 mg/kg body weight, and—for each dose—the course of and maximal change in other anterior pituitary hormone levels (GH, ACTH, TSH, LH, FSH and prolactin), IGF-1, fT4, cortisol and the change from baseline to end of test in plasma sodium, plasma osmolality and QTc-interval. Further, clinical symptoms during the tests were assessed and documented.

### Statistical analysis

Sample size was estimated in order to show an increase in copeptin level within two hours after the intake of a single oral-dose macimorelin. We used a resampling approach based on data of copeptin levels after arginine infusion from 50 healthy volunteers [4]. We assumed the absolute treatment effect of a single oral-dose macimorelin 0.5 mg/kg to be 0.5 pmol/L less than observed after arginine infusion. Hence, we expected a median change in copeptin of 2.3 pmol/L, as compared to 2.8 pmol/L. Sample sizes n_i_ = 10–50 were examined by sampling with replacement 999 times n_i_ data and testing them for a difference from zero at a significance level α of 0.05 using the Wilcoxon rank sum test. A sample size of 25 evaluable subjects would provide 90% power to reject the null hypothesis. Assuming a drop-out rate of 10% we aimed to recruit a total of 28 subjects.

Categorical variables are summarized by frequency (%) and continuous variables are summarized by mean (standard deviation; SD) in case of no obvious deviation from normal distribution or by median [interquartile range; IQR] otherwise. The maximum change in copeptin, the maximum changes in the other pituitary hormones and the changes in plasma sodium and osmolality after 0.5 mg/kg macimorelin and after 0.75 mg/kg macimorelin were each tested for a difference from zero using the sign test. The maximum change in copeptin was further compared for a difference between the two doses using the paired samples sign test. The within-subject differences in copeptin between the two doses are reported by median and IQR.

Since all these secondary analyses are exploratory, hypothesis-generating and not hypothesis-testing, no adjustment for multiple comparisons were made. P-values are not to be interpreted as confirmatory but as continuous measures that inform the generation of new hypotheses worthy of further investigation, in conjunction with the estimated effect sizes. In accordance with recent recommendations [14], the term “statistically significant” is not used.

All analyses were conducted using the statistical software package R (R Core Team, 2019), Version 3.6.0.

## Results

### Baseline characteristics

Twenty-eight healthy volunteers were included. They had a mean age of 22.4 years (SD 1.5), a median body mass index of 22.3 kg/m^2^ [IQR 21.5, 24.4] and 13 (46.4%) were male. A complete description of all baseline characteristics is provided in Table 1.

**Table 1:** Baseline characteristics are indicated as mean (standard deviation), median [inter-quartile range] or frequency (%) as appropriate

n	28
Male, n (%)	13 (46.4%)
Age, years (SD)	22.4 (1.5)
Alcohol consumption (glassweek)	2.0 [1.0, 3.0]
Current smoker, n (%)	0 (0%)
BMI (kg/m^2^)	22.3 [21.5, 24.4]
Oral contraceptive pill, n (%)	5 (33.3%)
Plasma sodium (mmol/L)	141 [140, 142]
Plasma osmolality (mmol/kg)	289.5 [288, 292]

Percentages for oral contraceptive pill refer to female participants

### Effect of macimorelin on copeptin levels and plasma osmolality

**Fig. 1:**
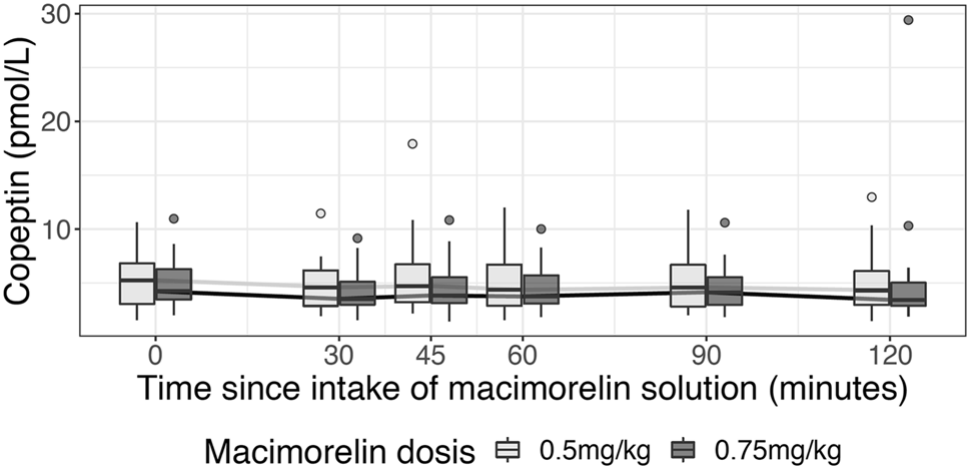
Time course of copeptin after a single oral stimulation with macimorelin 0.5 mg/kg and 0.75 mg/kg

**Fig. 2:**
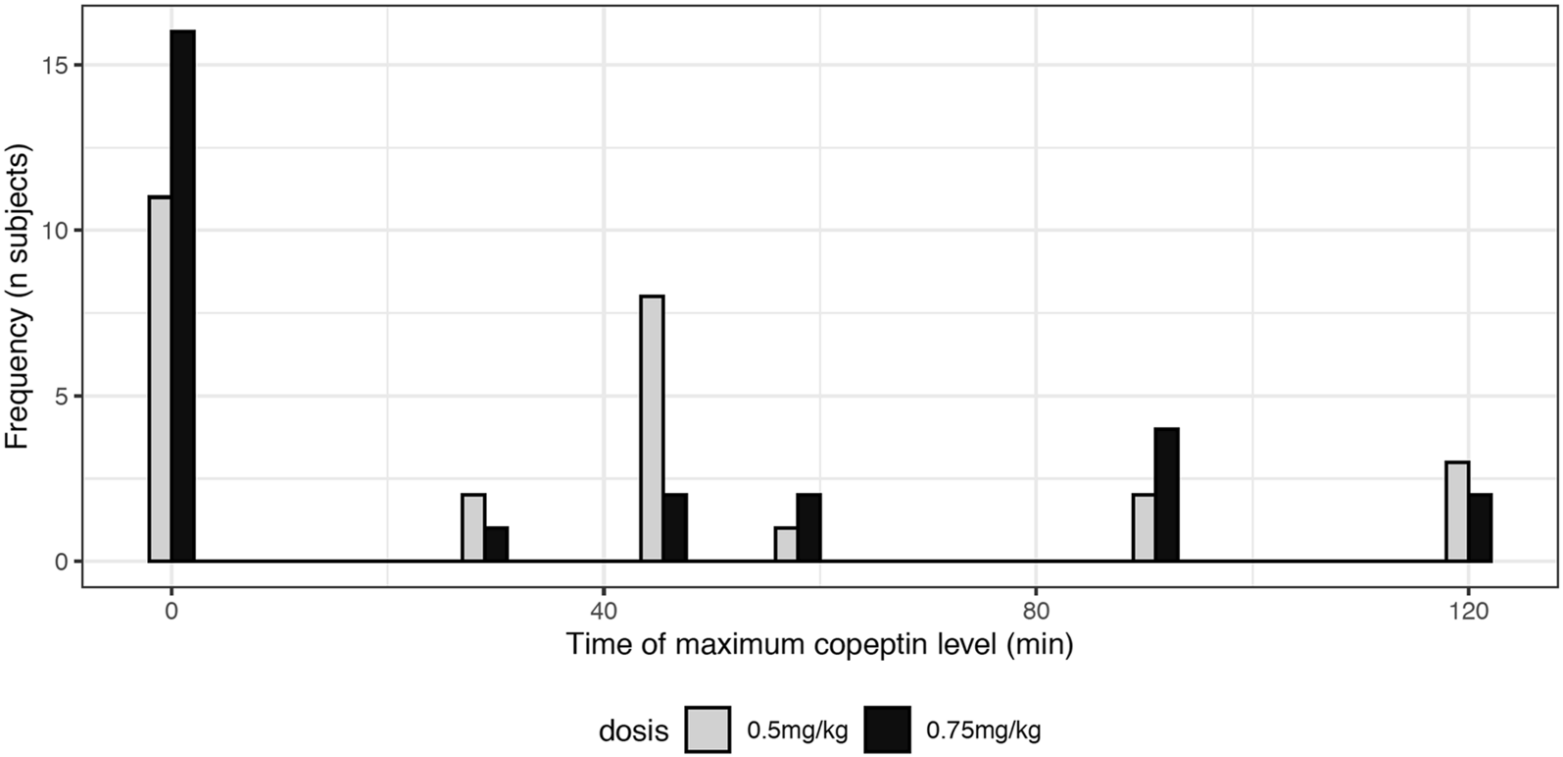
Maximum copeptin times, i.e. the measurement time point at which copeptin levels were maximal. Time zero indicates the baseline, i.e. before intake of macimorelin

Median [IQR] baseline copeptin levels were 5.26 [1.57, 6.81] pmol/L and 4.25 [2.04, 6.29] pmol/L for the 0.5 mg/kg and 0.75 mg/kg body weight doses, respectively. We found no evidence for an increase in copeptin for either dose (Fig. 1, Table 2). The maximal changes in copeptin levels were not different from zero: 0.40 [− 0.49, 0.65] pmol/L (p = 0.442) and − 0.13 [− 0.45, 0.17] pmol/L (p = 0.442), accordingly. For 11 and 17 subjects, respectively, all copeptin values after intake of macimorelin were even lower or equal to the respective baseline value. For those participants reaching their maximum level after the intake of macimorelin, the peak was most often seen after 45 min for the dose of 0.5 mg/kg and at one of the later time points for the dose of 0.75 mg/kg (Fig. 2, Table 2). The within-subject difference in maximal copeptin change between 0.75 mg/kg and 0.5 mg/kg macimorelin dose was not different from zero: − 0.21 [− 1.02, 0.42] pmol/L (p = 0.199). Plasma sodium levels or plasma osmolality slightly decreased (Table 2).

**Table 2:** Summary table of outcomes according to macimorelin dose

	Dose (mg/kg)	Baseline						Maximal changes						
		N	Minimum	First quartile	Median	Third quartile	Maximum	N	Minimum	First quartile	Median	Third quartile	Maximum	P value
Copeptin (pmol/L)	0.5	27	1.57	3.07	5.26	6.815	10.62	27	− 1.17	− 0.50	0.40	0.65	14.66	0.442
Copeptin (pmol/L)	0.75	27	2.04	3.48	4.25	6.295	10.97	27	− 1.61	− 0.45	− 0.13	0.18	21.36	0.442
GH (mlU/L)	0.5	28	0.16	0.435	3.665	13.325	40.9	28	− 3.51	56.50	94.67	110.96	137.81	< 0.001
GH (mlU/L)	0.75	27	0.15	0.31	1.6	17.25	58.4	25	6.43	80.40	101.10	128.20	147.69	< 0.001
IGF-1 (nmol/L)	0.5	28	19.7	25.975	30.95	38.625	45	28	− 1.60	− 0.30	0.65	2.33	5.10	0.185
IGF-1 (nmol/L)	0.75	27	21.9	26.05	30.1	38.35	43	27	− 0.80	− 0.10	0.40	1.10	3.60	0.442
fT4 (pmol/L)	0.5	28	11.1	14	15.45	16.25	17.6	28	− 1.00	0.20	0.50	0.80	1.50	< 0.001
fT4 (pmol/L)	0.75	27	9.5	13.6	14.8	16.5	18.3	27	− 0.30	0.40	0.60	0.90	1.50	< 0.001
TSH (mlU/L)	0.5	28	0.762	1.19	1.565	2.095	4.77	28	− 0.77	− 0.22	− 0.18	− 0.09	0.21	< 0.001
TSH (mlU/L)	0.75	27	0.685	1.405	1.72	2.33	4.25	27	− 0.97	− 0.32	− 0.17	− 0.07	0.10	< 0.001
Prolactin (mU/L)	0.5	28	136	245	303	417.25	675	28	− 214.00	2.50	100.00	146.50	339.00	0.004
Prolactin (mU/L)	0.75	27	131	236	312	429.5	694	27	− 113.00	12.50	101.00	309.50	640.00	< 0.001
Cortisol (nmol/L)	0.5	28	237	341.25	369	427.75	706	28	− 153.00	− 66.75	− 16.00	90.25	179.00	0.572
Cortisol (nmol/L)	0.75	27	174	307.5	379	429	766	27	− 157.00	− 58.00	− 14.00	55.50	346.00	0.701
ACTH (ng/L)	0.5	27	5	14.15	19.9	26.55	66	26	− 24.40	− 3.55	1.65	9.58	121.60	0.169
ACTH (ng/L)	0.75	27	6.5	14.05	18.6	24.85	56.8	27	− 13.30	− 0.85	2.10	8.60	102.40	0.248
LH (IU/L)	0.5	28	0.3	3.85	7.2	10.15	23.9	27	− 2.60	− 1.05	− 0.30	0.90	12.30	0.701
LH (IU/L)	0.75	27	0.3	3.9	5.1	6.95	16	26	− 2.70	− 0.60	− 0.25	1.23	6.30	0.557
FSH (IU/L)	0.5	28	0.3	3.225	4.75	5.475	8.3	27	− 0.40	− 0.10	0.00	0.20	1.40	0.442
FSH (IU/L)	0.75	27	0.3	2.5	3.8	5	8	26	− 0.50	− 0.10	0.00	0.20	0.50	0.327
Plasma sodium (mmol/l)	0.5	28	137	140	141	142	144	28	− 4.00	− 2.00	− 1.00	0.25	3.00	0.013
Plasma sodium (mmol/l)	0.75	27	138	139.5	141	141.5	145	27	− 4.00	− 2.50	− 2.00	0.00	5.00	0.006
Plasma osmolality (mmol/kg)	0.5	28	283	288	289.5	292	295	28	− 8.00	− 3.00	− 0.50	1.00	5.00	0.572
Plasma osmolality (mmol/kg)	0.75	27	284	288	290	292.5	299	27	− 9.00	− 3.00	− 2.00	0.00	7.00	0.002

### Effect of macimorelin on growth hormone levels

**Fig. 3:**
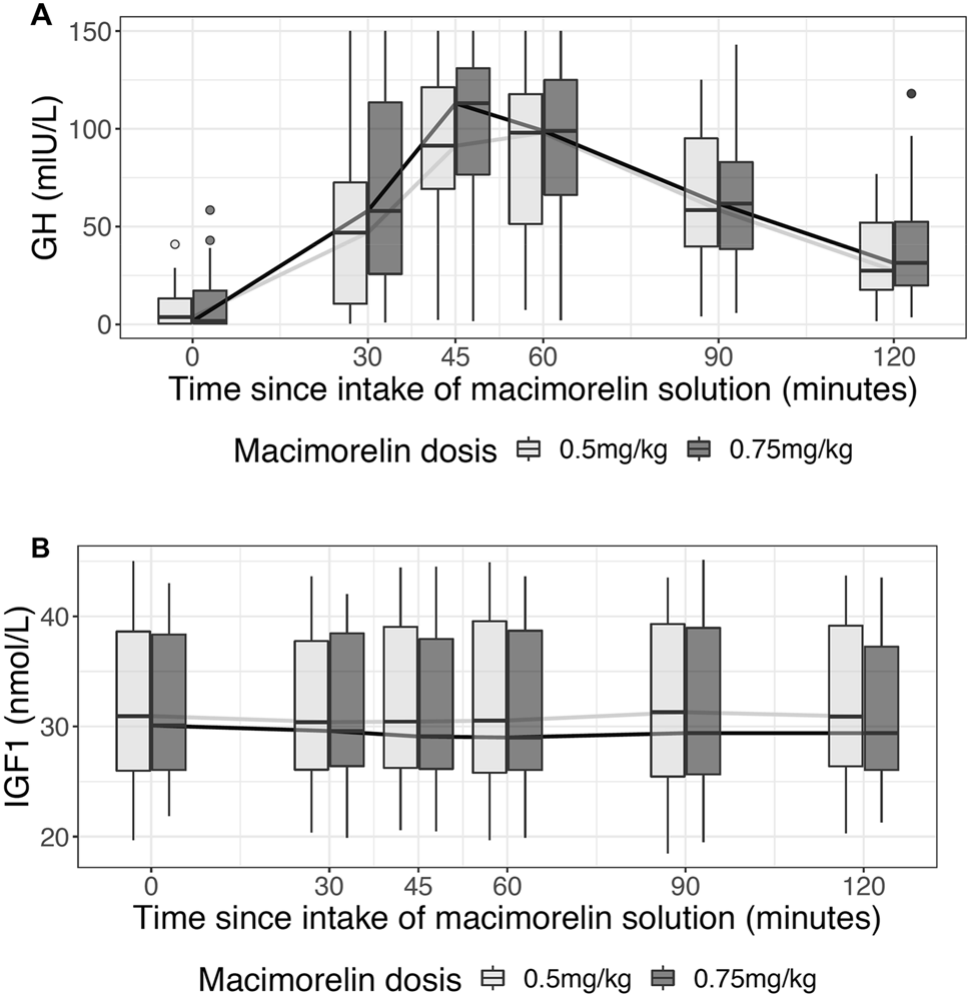
Time course of GH and IGF-1 for each macimorelin dose

Upon the intake of each single dose of macimorelin GH levels increased significantly (Fig. 3a, Table 2). The median [IQR] maximal change from baseline was 94.7 [56.5, 111.0] mU/L (p < 0.001) under the dose of 0.5 mg/kg body weight, and 101.1 [80.4, 128.2] mU/L (p < 0.001) under the dose of 0.75 mg/kg body weight. The peak was most often seen 45 min after the intake for either dose. However, we observed no change in IGF-1 levels upon intake of either dose (Fig. 3b, Table 2).

### Effect of macimorelin on the pituitary-adrenal axis

**Fig. 4:**
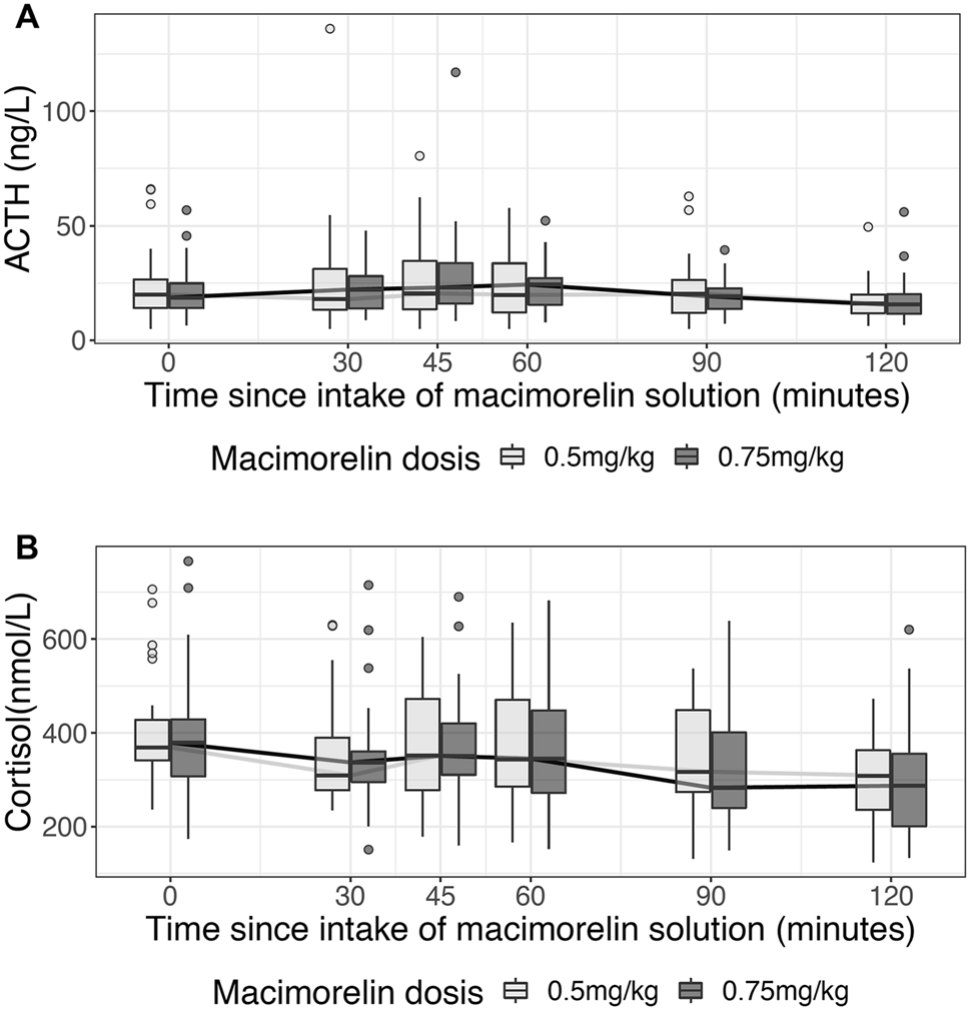
Time course of ACTH and cortisol for each macimorelin dose

Median ACTH levels at baseline were 19.9 [IQR 14.15, 26.55] pg/mL and 18.60 [14.05, 24.85] pg/mL for the 0.5 mg/kg and 0.75 mg/kg body weight doses, respectively. We found no evidence for an increase in ACTH levels for either dose (Fig. 4a, Table 2). The maximal changes in ACTH levels were not different from zero: 1.65 [− 3.55, 9.58] pg/mL (p = 0.169) and 2.10 [− 0.85; 8.60] pg/mL (p = 0.248), accordingly. Similarly, median baseline cortisol levels of 237 [341; 428] nmol/L did not change with a maximal median change of − 16.00 [− 66.75; 90.25] nmol/L (p = 0.572) after 0.5 mg/kg and − 14.00 [− 58.00, 55.50] nmol/L (p = 0.701) after 0.75 mg/kg macimorelin, respectively (Fig. 4b, Table 2).

### Effect of macimorelin on other anterior pituitary hormones and free thyroxine levels

**Fig. 5:**
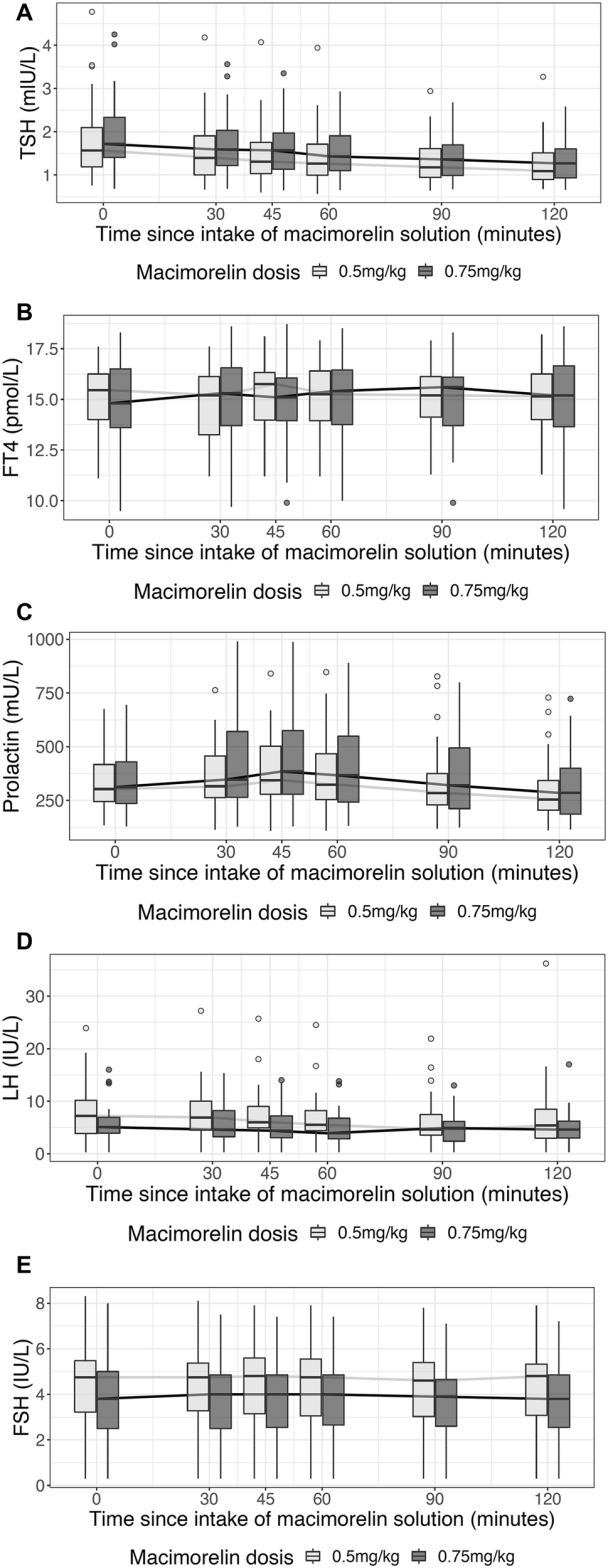
Time course of TSH, fT4, prolactin and gonadotropic hormones for each macimorelin dose

Median TSH levels decreased from 1.56 [IQR 1.19, 2.09] mU/L with a maximal median change of − 0.18 [− 0.22, − 0.09] mU/L (p < 0.001) after the dose of 0.5 mg/kg and a maximal median change of − 0.17 [− 0.32, − 0.07] mU/L (p < 0.001) after 0.75 mg/kg, respectively (Fig. 5a, Table 2). In contrast fT4 levels increased from a median at baseline of fT4 15.45 [14, 16.25] pmol/L with a maximal median change of 0.5 [0.2, 0.8] pmol/L, (p < 0.001) under the dose of 0.5 mg/kg and a maximal median change of 0.6 [0.4, 0.9] pmol/L (p < 0.001) under 0.75 mg/kg, accordingly (Fig. 5b, Table 2).

Prolactin levels increased at both doses from baseline levels 303 [245, 417] mU/L with a maximal median change of 100 [2.5, 146.5] mU/L (p = 0.004) after the dose of 0.5 mg/kg macimorelin. The maximal median change in prolactin levels after the higher dose of 0.75 mg/kg macimorelin accounted for 101 [13, 310] mU/L (p < 0.001) (Fig. 5c, Table 2). There was no change in LH or FSH levels in all study participants (Fig. 5d, e, Table 2) and also a subgroup analysis of men only did not reveal changes in LH or FSH levels.

### Safety and tolerability

There were no severe adverse events occurring during this study, the general tolerability was high and the recorded adverse events were mild: dizziness (4%), dysgeusia (4%), fatigue (18%) and headache (4%)***. ***Table 3 provides a full description of all adverse events during the study sequence including causality.

**Table 3:** Number of adverse effects occurring during the study sequence

Adverse effect	Dose (mg/kg)	Patients with event	Total patients	% affected patients
Diarrhea	0.5	0	28	0
Diarrhea	0.75	0	28	0
Dizziness^b^	0.5	1	28	4
Dizziness^b^	0.75	1	28	4
Dysgeusia^a^	0.5	1	28	4
Dysgeusia	0.75	0	28	0
Fatigue^b^	0.5	5	28	18
Fatigue^b^	0.75	3	28	11
Headache^b^	0.5	1	28	4
Headache^b^	0.75	1	28	4
Others	0.5	0	28	0
Others	0.75	0	28	0

^a^Causality of dysgeusia was probable

^b^All other adverse effects were assessed as unlikely to be related to the study drug macimorelin

The most relevant safety concern about macimorelin is QT-prolongation [13]. We noticed no QT prolongation during this study. The dose of 0.5 mg/kg macimorelin did not relevantly change median QT_c_ interval from baseline 405 [288, 411] ms to 412 [356; 422] ms after 120 min. Neither did the higher dose of 0.75 mg/kg macimorelin (QT_c_-interval_baseline_ 396 [391, 414] ms to QT_c_-interval_120 min_ 403 [391, 421] ms).

## Discussion

Our study has the following main findings. First, we found no evidence for a stimulatory effect of macimorelin 0.5 mg/kg or 0.75 mg/kg body weight on copeptin levels in healthy volunteers. Second, we confirm a stimulatory effect of macimorelin on GH levels in healthy volunteers. Third, we observed a stimulatory effect on prolactin and fT4, but not on ACTH nor on gonadotropic hormones.

The differential diagnosis of diabetes insipidus is important, as an incorrect diagnosis could result in an inadequate treatment and lead to serious consequences for the affected patients [2]. Copeptin measurement after hypertonic saline infusion is currently the test with the highest diagnostic accuracy for diabetes insipidus [3]. However, the test procedure is cumbersome as tight sodium monitoring is mandatory and discomfort as well as side effects occur often [1]. Ghrelin is known to potently stimulate vasopressin secretion in vitro [7, 8, 10] and in vivo in rodents [9]. Hexarelin a GHS was found to stimulate vasopressin in humans [15]. And so did arginine, another GHS, which was previously used to diagnose AGHD [16]. Arginine infusion has proven as a non-osmotic stimulation test for patients with diabetes insipidus with a comparable accuracy to the hypertonic saline infusion but a better tolerance [4]. Nevertheless, it still needs an intravenous application, making it an elaborative test procedure. By analogy to its GH-stimulating properties, an oral stimulation test with a single dose of macimorelin would be highly attractive to assess for the possibility of diabetes insipidus.

However, neither the standard dose of macimorelin 0.5 mg/kg used in AGHD testing nor 0.75 mg/kg did increase copeptin levels during the test sequence of 120 min. Copeptin levels were highest at baseline in many participants. For those reaching their maximum of copeptin levels after the intake of macimorelin, the peak was seen around 45 min after intake for the dose of 0.5 mg/kg, which correlated with the peak in GH levels. Our data are in line with a previous study investigating the effect of an acryl ghrelin infusion on thirst and copeptin levels in eight hypopituitary patients [17]. Also in this study no stimulatory effect was seen on vasopressin/copeptin levels.

We can only speculate why macimorelin in the investigated doses does not stimulate copeptin in contrast to arginine. In fact, we postulate a different pathway by which arginine stimulates the pituitary cells in contrast to macimorelin. A lack of effect due to insufficient bioavailability of macimorelin seems less probable for the following reasons. Firstly, because of the above mentioned study of Vestergaard et al. [17] in which also the intravenously administered ghrelin lacked to stimulate copeptin levels in humans. And secondly because macimorelin presented increased oral bioavailability when compared with other GHS [18].

One could argue that the absorption of macimorelin in our study was insufficient and higher doses are needed to successfully stimulate the release of copeptin. We did not measure plasma concentrations of the study drug, but previous studies found a single oral dose of macimorelin to induce a strong dose-dependent increase in GH levels lasting 120 min and peak plasma concentrations of the study drug were reached between 50 and 75 min [18, 19]. As the behaviour of GH stimulation in our study correlates well with former studies, we feel confident that macimorelin has been absorbed efficiently. At the time when this study was designed, we had based our hypothesis on the fact that the greatest GH-increases after macimorelin administration was observed with doses ranging between 0.5 and 1.0 mg/kg as we had considered a dose-response effect of macrimorelin on the GH receptors in the brain and possibly on copeptin secretion. However, our present study refuted that hypothesis that there was a dose–response effect of macimorelin on copeptin secretion. More recently, Klaus et al. [20] reaffirmed the fact that macrimorelin dose range between 0.5 and 1.0 mg/kg provided the best dose range to induce GH secretion and transient increases in ACTH, cortisol, and prolactin levels, which were not macimorelin dose-related. However, whether even higher doses of macimorelin > 0.75 mg/kg are needed to observe increases in copeptin levels remains unclear. The use of supra-physiological doses of macimorelin up to 2 mg/kg body weight has been reported to be safe with no significant adverse events [13, 20] and therefore further evaluation with higher doses might be interesting. We conclude that neither the standard dose of 0.5 mg/kg body weight nor the higher dose of 0.75 mg/kg macimorelin have stimulatory effects on copeptin and therefore cannot be recommended to be used for the differential diagnosis of diabetes insipidus.

Macimorelin acetate effectively stimulates endogenous GH secretion in healthy volunteers [19] and has only recently proven as an accurate, well-tolerated and safe diagnostic test for patients with AGHD [6]. Our data confirm the stimulatory effect of macimorelin on GH levels and are in line with a previously published dose-escalation study in healthy volunteers [19], where doses of 0.05 mg/kg up to 0.5 mg/kg stimulated GH secretion. The maximal change of GH after the dose of 0.5 mg/kg was similar in our study compared to the mentioned study [19]. As reported earlier, we confirm that the stimulatory effect of macimorelin is limited to GH without any stimulation of IGF-1 levels [5]. This is probably explained by the longer half-life of IGF-1 levels compared to half-life of GH levels [21].

We also confirm the high tolerability and safety profile of macimorelin. The study drug was well tolerated and no severe adverse events occurred. A QT-prolongation of about 11 ms has been described during the development of macimorelin [13]. One single participant of a former study [6] had an asymptomatic QT-prolongation. However, in this case there was described a co-medication with citalopram, which is known to cause QT prolongation. We noticed no prolongation of the QT-interval in our study. Our data are therefore reassuring concerning the safety and tolerability profile of macimorelin used in this new diagnostic test for AGHD.

The reference standard for diagnosing ACTH deficiency is the ITT, an elaborate and for some patients an unpleasant and risky test procedure due to the induced hypoglycemia. Therefore, also for adrenal insufficiency, an easier to perform, less risky and orally administered stimulation test would be highly attractive. Besides the already discussed stimulation on GH, GHS also stimulate other anterior pituitary hormones such as prolactin, ACTH and cortisol. Hexarelin as one of the most potent GHS has been found to stimulate the HPA-axis in 15 healthy young males [11]. However, Hexarelin did not prove as a useful test for the evaluation of ACTH/cortisol reserve [15] as it could not consistently discriminate between patients with pituitary insufficiency from healthy controls as it did not stimulate cortisol to a level, which could rule out adrenal insufficiency [15]. The effects of macimorelin on the HPA-axis have been investigated only in few healthy volunteers (n = 9 and n = 6) with doses of 0.5 mg/kg body weight showing inconsistent responses of ACTH and cortisol [19, 20]. In our study including a larger sample size, we observed no increase neither in cortisol nor in ACTH level, but cortisol levels rather decreased following the circadian rhythm. Macimorelin in the investigated doses therefore cannot be recommended as a test for diagnosing adrenal insufficiency.

In contrast, macimorelin stimulated prolactin levels with an increase with peak values around 45 min, correlating with GH-peaks. This is in agreement with previous studies [11].

GHS-receptors are also found in the thyroid gland [22]. In rats, thyroxine (T4) and TSH levels decreased upon central injection of ghrelin [23, 24]. In healthy volunteers, no effect of macimorelin was found on TSH levels [20]. Interestingly and in contrast to this study, we observed a significant decrease in TSH levels, consistent with the findings in animal studies, and vice versa an increase in fT4 levels, thus leading to a constellation towards “hyperthyroidism”. To the best of our knowledge, this has not yet been described and is a novel and physiopathologically interesting finding. No stimulatory effect was found on LH and FSH levels.

Our study has several strengths and limitations. Strengths are that it is the first study investigating the effects of macimorelin on posterior pituitary hormones, particularly copeptin/vasopressin. Furthermore, we investigated our hypothesis with a concise study design, sample size consideration and sound methodology. As a limitation, we did not use a placebo controlled study design, as this was a proof-of concept study. However, by confirming the results in GH stimulation, we have a positive control and are confident that our results can be considered as valid and reliable. A further limitation is that IGF-1 levels were measured by LIAISON XL, as it is known that this assay can be affected by IGF-binding proteins.

To conclude, we herein confirm the potent stimulatory effect of macimorelin on GH release in healthy volunteers. However, macimorelin did not stimulate copeptin levels and therefore cannnot be used as a simple oral test for the differential diagnosis of diabetes insipidus. Additionally, macimorelin also exerted a stimulatory effect on prolactin and thyroid hormones, but not for cortisol, ACTH or gonadotropic hormones.

## Data Availability

The datasets generated during and/or analysed during the current study are available from the corresponding author on reasonable request.
